# Oil Things Bright and Beautiful? How Hydrocarbon Pollution Impacts Guppy Ornamentation

**DOI:** 10.1002/ece3.73105

**Published:** 2026-02-18

**Authors:** Hannah Rose McGovern, Francesco Santi, Amy Deacon, Rüdiger Riesch

**Affiliations:** ^1^ Department of Biological Sciences, Royal Holloway University of London Surrey UK; ^2^ Department of Life Sciences The University of the West Indies St Augustine Trinidad and Tobago

**Keywords:** carotenoids, evolution, freshwater ecology, pigmentation, *Poecilia reticulata*, sexual selection, the Pitch Lake

## Abstract

Brightly coloured male ornamentation often plays an important role in sexual selection, but the extent to which the expression of these ornaments is affected by different forms of pollution is still not well understood. Guppies ( *Poecilia reticulata* ) can often be found in highly polluted and degraded habitats, including oil‐polluted habitats in southwestern Trinidad. Male guppy ornamentation is polymorphic, and while colour patterns (including area cover of different ornaments) have a heritable basis, the intensity of different colour patches can be linked to phenotypic plasticity, partially modulated via guppy diet. Here, we aimed to understand how the area and colour intensity of this ornamentation varied between the Pitch Lake (a natural source of crude‐oil pollution), two anthropogenically polluted and two non‐polluted sites. We found that colour intensity and area of ornamentation both differed between habitat types, with males from anthropogenically polluted habitats having a greater area of iridescence ornamentation but darker orange colour intensity. However, for both area and intensity, there was considerable variation between polluted populations. Additionally, ornamentation in the Pitch Lake differed from both anthropogenically polluted and non‐polluted habitats. This study demonstrates the non‐uniform response to crude oil pollution even on a species level, and highlights the importance of considering both evolutionary and ecological variation when predicting and mitigating impacts of pollution.

## Graphical abstract

Spatial variation in crude oil pollution in Trinidad leads to differing levels of pollutant exposure in connected guppy populations. Male guppy ornamentation is an important sexually selected trait affected by environmental changes. Measuring area and intensity of this ornamentation, we found guppies from anthropogenically polluted habitats had greater area of iridescence and darker orange intensity compared to non‐polluted habitats. The naturally polluted Pitch Lake had a unique phenotype compared to both anthropogenically polluted and non‐polluted habitats.

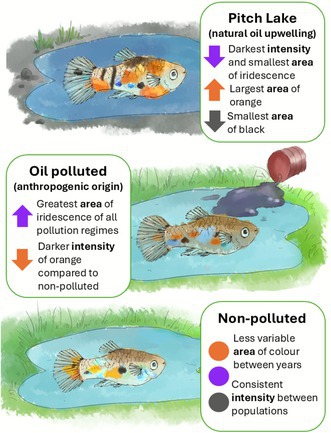

## 1 Introduction

Globally, freshwater habitats are considered some of the most at‐risk ecosystems, with freshwater vertebrate populations showing an 80% decline in the last 50 years—twice that of terrestrial or marine systems (Darwall et al. 2018). One of the major factors driving the decline in these ecosystems is anthropogenic pollution (Dudgeon et al. 2006). Crude oil is a major source of environmental pollution and can enter freshwater habitats through spills and contamination from oil field exploitation, oil refinery effluent, and resource transportation (Kelly et al. 2010; Wake 2005). Impacts of crude oil pollution include mortality (Freedman 1995), reduced fitness and developmental deformities in affected organisms (Incardona et al. 2013; Rowe et al. 1983; Sumpter 2009), as well as alterations in community composition (Olsgard and Gray 1995) and decreased population density.

Crude oil exploitation has been carried out in Trinidad and Tobago for over 100 years, with onshore exploitation occurring in the south of Trinidad (Government of the Republic of Trinidad and Tobago 2024). This has led to high levels of chronic crude oil pollution within waterways in the south of the island; however, this pollution is spatially varied, as tributaries within the same drainage system can range from highly polluted to essentially non‐polluted (Rolshausen et al. 2015; Santi et al. 2021). In addition to spillage from the oil industry, another source of crude oil contaminants is the Pitch Lake, a naturally occurring asphalt lake containing many of the same compounds found in anthropogenic oil pollution including polycyclic aromatic hydrocarbons (PAHs) (Ponnamperuma and Pering 1967; Santi et al. 2021). Unlike most polluted habitats, the Pitch Lake is a well‐established, biodiverse system that sustains several different plant, fish, bird, and amphibian species (Mohammed et al. 2010; Schelkle et al. 2012; Santi et al. 2019).

Despite the lethal and sub‐lethal impacts of crude oil on aquatic organisms, live‐bearing guppies ( *Poecilia reticulata* , family Poeciliidae) are able to tolerate high levels of pollution and even maintain large populations in polluted environments (Araújo et al. 2009; Gomes‐Silva, Cyubahiro, et al. 2020; Rolshausen et al. 2015). In Trinidad, guppies have independently colonised crude oil‐polluted waterways, as well as the Pitch Lake (Rolshausen et al. 2015; Santi et al. 2019, 2021). However, the impacts of this exposure to crude oil pollution on affected populations are not well understood. Morphological studies found contrasting results, with Rolshausen et al. (2015) initially finding guppies from polluted habitats had longer and shallower bodies, whilst a more recent investigation by Santi et al. (2021) found that guppies from polluted habitats were larger than those from neighbouring non‐polluted habitats, with rounder and deeper bodies. This incongruity in results suggests responses to pollution are not consistent across populations and may even vary across years within the same populations.

Male ornamentation in the form of skin colouration is an important trait in guppy mate choice, and has been extensively studied as a model to understand patterns of sexual selection (Endler 1991; Godin and McDonough 2003; Houde 1997). There are three main colouration types: black, formed by melanin pigments; orange/red, formed by carotenoids and pteridines; and iridescence colours formed by structural formations of guanine crystals (Grether, Hudon, and Endler 2001; Kottler et al. 2014). These colouration traits are highly polymorphic (Endler 1983). The location and area size of pigmentation have a strong genetic basis (Winge and Ditlevsen 1947; Houde 1992), whilst the intensity of the colour patches, particularly orange and black, is more plastic, strongly dependent on diet and, thus, can fluctuate within an individual's lifetime (Grether, Hudon, and Endler 2001; Kodric‐Brown 1985; Endler 1983). This has enabled the capacity for rapid divergence of ornamentation traits in response to different environmental conditions, including predation regime (Endler 1991; Kemp et al. 2008), turbidity (Camargo‐Dos‐Santos et al. 2021), and canopy cover (Grether, Millie, et al. 2001). It is expected therefore that ornamentation will also diverge in response to the physiological and environmental pressures caused by crude oil pollution.

Crude oil pollution has the potential to directly hamper ornamentation development through the effects on immune system functioning. In birds, experimental exposure to crude oil led to a decrease in the size of the red bill spot as carotenoids were allocated to enhance immune functioning and PAH degradation (Pérez et al. 2010). Carotenoids are an important pigment in guppy ornamentation, and there is an observed trade‐off between allocation of carotenoids for immune function and orange ornamentation (Houde and Torio 1992). It is possible therefore that exposure to oil pollution will reduce the amount of carotenoids available for ornamentation and that guppies from polluted habitats will subsequently have reduced amounts and intensity of orange colouration.

In addition to altered immune functioning, disordered embryonic development may have knock‐on effects on secondary sexual development, impacting growth of pigment cells in adult guppies. Dark‐edged splitfin fish ( *Girardinichthys multiradiatus* ) exposed to chemical pollutants had lower yellow chroma as adults due to defects during embryonic development (Arellano‐Aguilar and Garcia 2008). Crude oil pollution is known to affect embryonic development in fish (Incardona et al. 2013), and therefore it is possible that these developmental defects will affect ornamentation growth as adults, reducing area and intensity of colour patches.

Besides direct impacts of crude oil pollution on pigment allocation, indirect effects via changes to community ecology may also alter ornamentation. Crude oil pollution is known to reduce productivity which may reduce availability of the unicellular algae from which guppies derive most of their dietary carotenoids. This reduces colour intensity and inhibits development of orange ornamentation (Grether et al. 1999). Crude oil pollution may also affect the predation regime. If predatory fish are less tolerant of pollution than guppies, then polluted habitats may act as a refuge from predation (Gomes‐Silva, Pereira, et al. 2020). Guppies are known to rapidly adapt to a release from predation pressure, increasing the area of ornamentation (Endler 1991). One study of guppies exposed to urban and agricultural pollution found guppies from polluted habitats had more orange, blue, and black ornamentation, which may suggest a release from predation pressure (Gomes‐Silva, Cyubahiro, et al. 2020). It is likely therefore that several secondary environmental effects of oil pollution may also affect ornamentation patterns.

Here we aim to investigate how oil pollution affects guppy ornamentation and if the source of oil pollution (natural vs. anthropogenic) is important. To address this we sampled in five different populations, three representing oil‐polluted habitats (the naturally polluted Pitch Lake and two anthropogenically polluted sites) and two non‐polluted habitats. We made three predictions about the impacts of oil pollution on male guppy ornamentation: (1) Pollution will lead to a decrease in orange colouration, due to a reduction in available dietary carotenoids and increased burden on the immune system requiring allocation of carotenoids away from ornamentation development. (2) Black and iridescent ornamentation will increase in polluted populations to compensate for a lack of orange and in response to a reduction in predation pressure; although it is also possible that the previously mentioned impact of oil on development might hinder the expression of structural colours, which would then result in reduced iridescence. (3) Males from the Pitch Lake population will not follow the same trends of ornamentation differences as seen in anthropogenically polluted populations due to the unique ecology of the Pitch Lake.

## 2 Material and Methods

### 2.1 Study Sites

**FIGURE 1:**
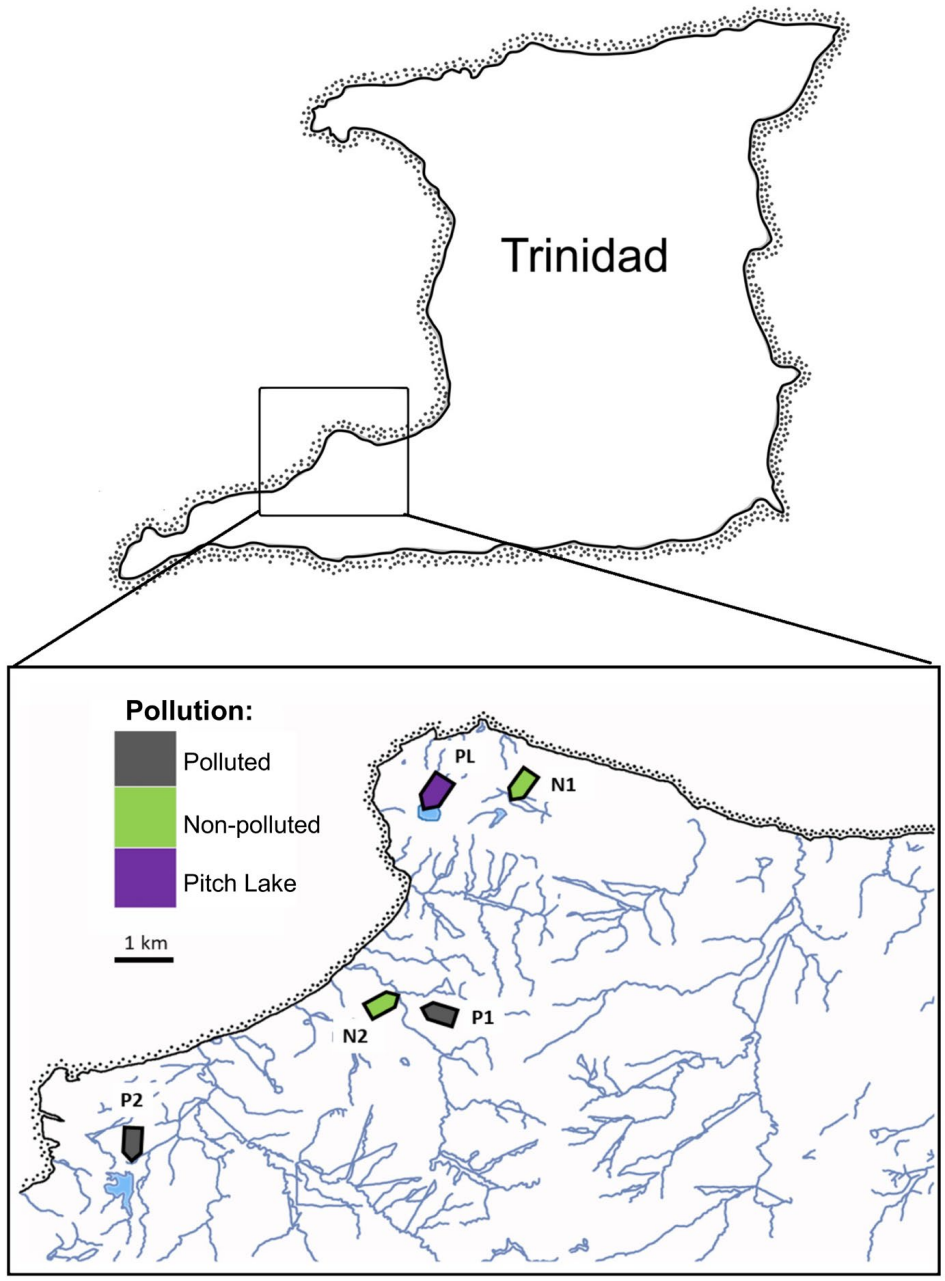
Map of study sites sampled in 2018 and 2019. Anthropogenically polluted sites are indicated in grey, the Pitch Lake in purple, and non‐polluted sites are indicated in green. Maps created by R. Riesch; drainages in the inset were extracted from www.caribbeanmarineatlas.net.

We collected guppies in five sites across southern Trinidad in May/June 2018, and again in May/June 2019 from a subset of those studied by Santi et al. (2021) (Figure 1). The first site was the Pitch Lake (PL), a natural asphalt lake resulting from upwelling of bitumen and crude oil (including PAH). Further, we sampled two sites subject to anthropogenic crude‐oil pollution: a small stream from the Vance River drainage (P1) and a drainage ditch in the town of Point Fortin (P2). As reference sites, we included two sites with no evidence of oil pollution (including no discoloration on Macherey‐Nagel Oil test strips), one close to the Pitch Lake (N1) and one in the Vance River drainage (N2) (Figure 1; see Santi et al. 2021 for more information).

Using a Hach Rugged Field Kit (Hach, Loveland, Colorado, USA) we further measured water temperature [°C], dissolved oxygen [mg/L], salinity [ppt], conductivity [microS/cm] and pH. Barring the Pitch Lake (PL), all sites were similar in structure, being low flow, shallow (30–60 cm) drains with a combination of concrete, rubble and mud substrate and width of 1–3 m. The Pitch Lake, however, is around 100 acres in size with variable depth from between 20 cm to over 1.5 m in the permanent pools around the edge of the site. In addition to abiotic measurements, any potential aquatic guppy predators that were observed during sampling were also identified (Table S1).

We collected all fish using hand‐held seines and dip nets, then transported them to the laboratory at The University of the West Indies at St. Augustine, where they were housed in identical, mixed‐sex 100 L tanks and allowed to settle for 48 h. All tanks were aerated and kept under natural sunlit conditions (no artificial lighting) in tanks heated only by ambient air temperature, maintaining a water temperature of around 27°C during the day. Fish were fed *ad libitum* twice daily with commercial flake food (TetraMin flakes, Tetra).

### 2.2 Ornamentation Area and Number of Spots

In the laboratory after 48 h, males were randomly selected from the 100 L holding tanks and anesthetised using clove oil (Fernandes et al. 2017). We then immediately photographed the left side of each male—along with a scale for calibration—using a DLSR camera (Canon EOS 7D Mark II, with a 35 mm macro lens; Canon inc., Tokyo, Japan) mounted on a copy stand under standardised light conditions. A minimum of 10 and up to 20 males were photographed per population each year (Table S1). Representative photographs illustrating highest and lowest area and intensity colour values are included in the appendix (Figure S1). Photos were then analysed using ImageJ (https://imagej.net/; Schneider et al. 2012), measuring Standard Length (SL [mm]), total body area, and the number and area of differently coloured spots. This analysis was carried out by the same researcher to prevent observer bias. Each spot was measured by outlining it using the polygon tool in ImageJ (Ruell et al. 2013). We thus measured the total area of orange/red colouration (henceforth Orange), the total area of blue, green, silver and violet structural colour spots (Iridescence), and the total area of black spots for each male (Cattelan et al. 2020; Devigili et al. 2015). The total area covered by each colour was then divided by total body area of that fish (also measured using imageJ) to obtain percentage body cover for all three colouration types.

### 2.3 Colour Intensity

Measurements of colour intensity were taken using MicaToolbox (Troscianko and Stevens 2015), a plugin for ImageJ that enables RGB formatted images to be converted into CIELAB space such that L*a*b* values can be quantified as a measure of colour intensity, through values of lightness (*L*), green‐to‐red (*a*), and blue‐to‐yellow (*b*). Photographs were linearised using MicaToolbox to ensure pixel values scaled with light intensity, and then standardised using a ‘cone‐catch model’ that creates absolute colour values based on spectral‐reflectance curves for a diffuse colour chart (Greywhitebalance colour card CT28 in this instance) that was photographed under the same conditions. Unfortunately, there was no photograph taken of the diffuse colour chart in 2019, so to avoid any pitfalls resulting from discrepancies in lighting or camera set up affecting standardisation, we did not include 2019 photographs in our analysis on colour intensity. However, we have included the results of the 2019 analysis in a Supporting Information (Table S2) for posterity.

After using MicaToolbox to create CIELAB images of initial photographs, L*a*b* values were measured in much the same way as area. Each coloured spot was outlined using the polygon tool in ImageJ and the L*a*b* values measured. These then were averaged for each individual fish across the three colour types to generate (in the case that all three colours were present) L*a*b* values per colour for each fish. Following Mokrzycki and Tatol (2011), the three L*a*b* colour spaces were then combined to one value, Δ*E*, which expresses total differences in colour. Δ*E* was calculated by determining the maximum value for each measure of colour space (L*, a* and b*) within each colour type (orange, iridescence and black), and then applying the following formula:

$$\begin{array}{cc} \Delta E\;=\; & \text{square root}[{\left(L_{\text{observed}}^{*}-L_{\text{maximum}}^{*}\right)}^{2}\\ & +{\left(a_{\text{observed}}^{*}-a_{\text{maximum}}^{*}\right)}^{2}+{\left(b_{\text{observed}}^{*}–b_{\text{maximum}}^{*}\right)}^{2}] \end{array}$$

This calculates the difference in each colour type between individual fish and the most extreme colour value.

### 2.4 Statistical Analysis

Statistical analyses were conducted using IBM SPSS Statistics version 28.0.1.1 (IBM Corporation, Armonk, New York, USA). Resulting data was plotted using R Statistical Software (v4.4.2; R Core Team 2024) with the ggplot2 package (v3.3.3; Wickham 2016).

As the area and number of spots of ornamentation are dictated primarily by genetic factors, whilst colour intensity is a variable, plastic trait (Endler 1983; Grether, Hudon, and Endler 2001), we analysed these traits separately as the underlying drivers and, therefore, effects of pollution may be different.

For analysing area of ornamentation, we first carried out factor reduction via principal component analysis (PCA) with Varimax rotation on the number of spots and percentage of surface area coverage for each of the colour categories. We decided to extract three PCs, though the third only had an eigenvalue of 0.99. However, these three PCs, which cumulatively explained 70.52% of the total variance (Table 1), represented a clear separation of the three different colour types: orange, black and iridescence. Specifically, the number of spots and percentage surface area for orange loaded onto PC1, the number of spots and percentage surface area for iridescence loaded onto PC2, and the number of spots and percentage surface area for black loaded onto PC3. In addition, a moderate amount of variance for the number of black spots also loaded onto PC2 (Table 1).

**TABLE 1:** Results of factor reduction using a Varimax‐rotated principal component analysis (PCA) on area of male body ornamentation. Eigenvalues and percentage of variance explained by the three extracted components are listed.

	Principal component		
	1	2	3
No. orange spots	0.870	—	—
Percent orange	0.839	—	—
No. iridescent spots	—	0.811	—
Percent iridescence	—	0.791	—
No. black spots	—	0.428	0.669
Percent black	—	—	0.856
Eigenvalue	1.775	1.466	0.990
Percentage of variance explained	29.579	24.439	16.505
Cumulative percentage of variance	29.579	54.017	70.522

To test for differences in area of ornamentation between pollution regime (Pitch Lake vs. anthropogenically polluted vs. non‐polluted), between populations within pollution regime as well as for temporal differences between years (2018 vs. 2019), we then carried out a multivariate Analysis of Covariance (MANCOVA) using all three PCs with pollution regime, year and ‘population nested‐within pollution regime’ [henceforth: population(pollution)] as independent variables, while also including male standard length as a covariate. The assumption of equality of error variances was met for all three PCs (Levene's test; PC1: *F =* 0.676, *p =* 0.73; PC2: *F =* 1.331, *p =* 0.225; PC3: *F =* 1.323, *p =* 0.229), and the assumption of equality of covariance was also met (Box's M test: *F =* 1.052, *p =* 0.370). Nonetheless, to be consistent with our other analyses (see below), we evaluated significance based on Pillai's Trace in our MANCOVA. To further explore any significant effects on individual dependent variables, we then applied post hoc ANCOVAs of the same structure.

For colour intensity we used Δ*E* values as dependent variables. All colour types were normally distributed in 2018. A MANCOVA was used that only included pollution regime and population (pollution) as independent variables and standard length as a covariate. The assumption of equality of covariance was not met (Box's *M* test: *F =* 1.874, *p =* 0.006), nor was the assumption of equality of error variances for black (Levene's test; black: *F* = 2.557, *p =* 0.046). Therefore, we evaluated significance based on Pillai's Trace in our MANCOVAs (Bray and Maxwell 1985). Post hoc ANCOVAs were also carried out on each colour type using the same structure as the MANCOVA.

### 2.5 Repeatability

All measurements were made by one observer to prevent observer bias. To test for repeatability, 5 fish per population were selected at random and the number of spots and area of ornamentation were re‐measured by the same observer. The intra‐rater reliability of number of spots was measured using Cohen's kappa. For all three colour types, kappa ranged from 0.617 to 0.780, indicating good repeatability (Altman 1999). Area of ornamentation was evaluated using intraclass correlation coefficients (ICCs), and all measures had good to excellent reliability (ICCs ranged from 0.755 to 0.952; Koo and Li 2016). All repeatability analyses were conducted in IBM SPSS Statistics version 28.0.1.1 (IBM Corporation, Armonk, New York, USA).

## 3 Results

### 3.1 Number of Spots and Body Area of Ornamentation

In the MANCOVA, the covariate standard length had no significant influence on area of ornamentation (*p =* 0.197), nor was there significant variation across populations within pollution regime (nested effect: *p* = 0.454). There were, however, significant differences between pollution regimes (*p* < 0.001). In addition, there was a non‐significant trend between years (*p =* 0.077), as well as a non‐significant trend for the interaction effect of pollution regime‐by‐year (*p* = 0.067), but no effect of population(pollution)‐by‐year (*p* = 0.638) (Table 2A).

**TABLE 2:** Results of MANCOVAs comparing (A) effects of year, pollution, population nested‐within pollution [population(pollution)] and interaction effects on area of ornamentation with standard length as a co‐variate. (B) Effects of pollution and 'population nested‐within pollution [population(pollution)] on intensity of colouration (Δ*E*) for 2018 with standard length as a co‐variate. Significant *p‐*values are highlighted in bold, and effect size was estimated via partial eta squared ($\eta_{p}^{2}$).

Factor	*F*	df	*p*	$\eta_{p}^{2}$
(A) Area of ornamentation				
Standard length	1.621	3151	0.187	0.031
Year	2.327	3151	0.077	0.044
**Pollution**	**6.741**	**6304**	**< 0.001**	**0.117**
Population(Pollution)	0.957	6304	0.454	0.019
Year × Pollution	1.987	6304	0.067	0.038
Year × Population(Pollution)	0.7141	6304	0.638	0.014
(B) Colour intensity (Δ*E*)				
Standard length	1.284	3,71	0.287	0.051
**Pollution**	**3.976**	**6144**	**0.002**	**0.136**
**Population(Pollution)**	**3.1512**	**6144**	**0.007**	**0.115**

**FIGURE 2:**
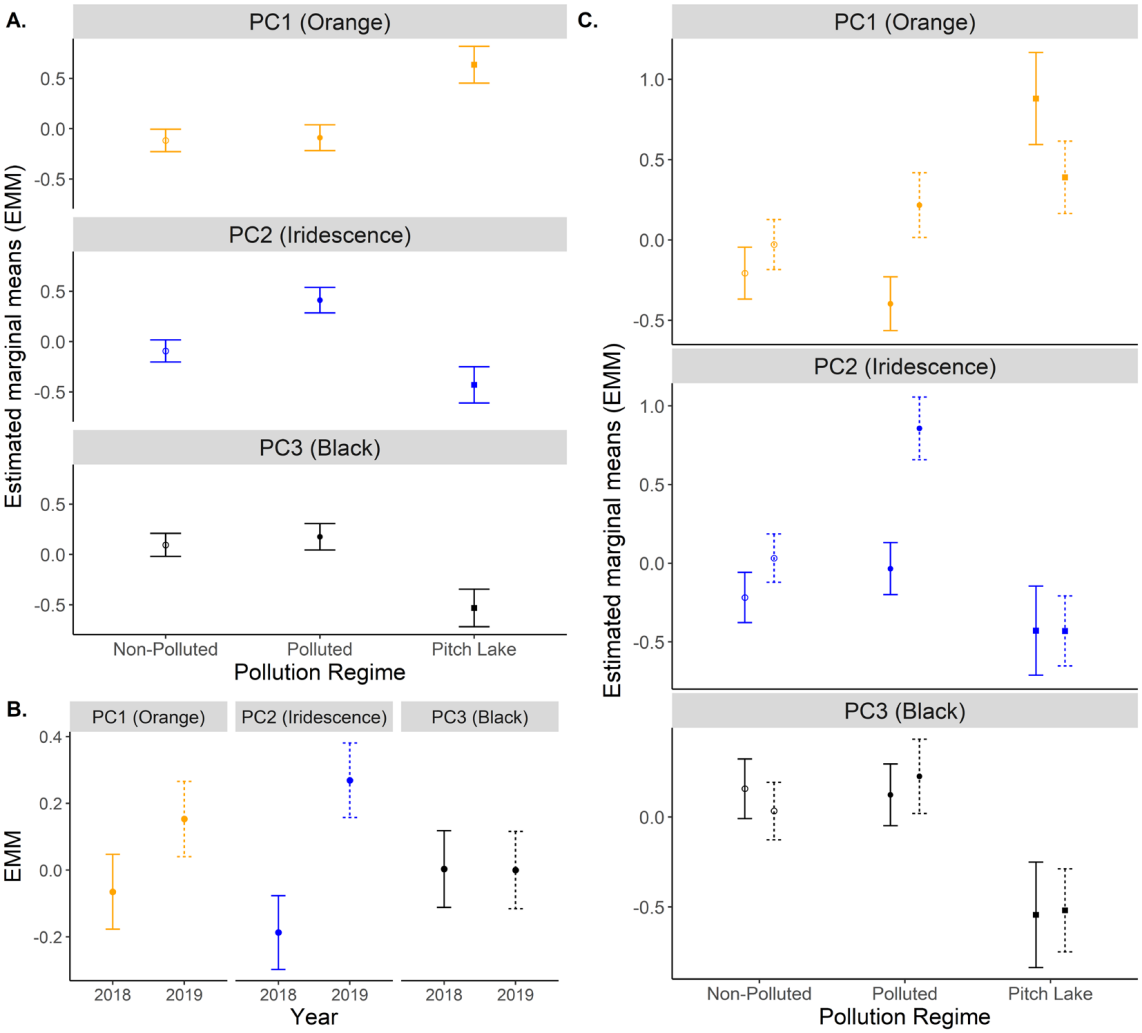
Estimated marginal means (±SE) for area of ornamentation (A) across pollution regimes, (B) between years, and (C) between years and pollution regimes. Pollution regime indicated by shape, with open circle denoting ‘non‐polluted’, closed circle as ‘anthropogenically polluted’ and closed square as ‘Pitch Lake’. Year indicated by line type, with 2018 as solid and 2019 as dashed.

In our post hoc ANCOVAs (Table 3A), neither standard length nor population(pollution) had significant effects on any individual PC, although there was a non‐significant positive trend for iridescence (PC2: *p =* 0.075). There were, however, significant effects of pollution regime on all PCs (PC1: *p =* 0.002; PC2: *p <* 0.001; PC3: *p =* 0.006; Table 3A). Post hoc pairwise comparisons of estimated marginal (EM) means between pollution regimes found that for iridescence (PC2), anthropogenically polluted habitats differed from both the Pitch Lake and non‐polluted habitats, while there was no significant difference between Pitch Lake and non‐polluted habitats. For both orange (PC1) and black (PC3), however, there was no difference between anthropogenically polluted and non‐polluted habitats, but the Pitch Lake differed significantly from both (Table 4A, Figure 2A).

**TABLE 3:** Results of post hoc ANCOVAs on (A) the individual principal components (PCs) representing area of ornamentation and testing for the effects of year, pollution, ‘population nested‐within pollution’ [population(pollution)] and interaction effects. Standard length was also included as a covariate. (B) Effects of pollution and population(pollution) on intensity of ornamentation (Δ*E*) in 2018 with standard length as a covariate. Significant *p‐*values are highlighted in bold.

Dependent variable	Factor	*F*	df	*p*
(A) Area of ornamentation				
PC1 (Orange)	Standard length	0.046	1152	0.83
	Year	0.842	1152	0.360
	**Pollution**	**6.717**	**1152**	**0.02**
	Population(Pollution)	0.339	1152	0.713
	**Year × Pollution**	**3.177**	**1152**	**0.044**
	Year × Population(Pollution)	1.318	1152	0.271
PC2 (Iridescence)	Standard length	3.209	1152	0.075
	**Year**	**6.217**	**1152**	**0.014**
	**Pollution**	**8.453**	**1152**	**< 0.001**
	Population(Pollution)	0.889	1152	0.413
	Year × Pollution	2.746	1152	0.067
	Year × Population(Pollution)	0.162	1152	0.850
PC3 (Black)	Standard length	1.383	1152	0.241
	Year	0.00	1152	0.999
	**Pollution**	**5.211**	**1152**	**0.006**
	Population(Pollution)	1.760	1152	0.175
	Year × Pollution	0.216	1152	0.806
	Year × Population(Pollution)	0.845	1152	0.432
(B) Colour intensity				
Δ*E* Orange	Standard length	1.631	1,73	0.206
	**Pollution**	**3.92**	**2,73**	**0.024**
	**Population(Pollution)**	**6.315**	**2,73**	**0.003**
Δ*E* Iridescence	Standard length	0.148	1,73	0.702
	**Pollution**	**5.536**	**2,73**	**0.006**
	**Population(Pollution)**	**4.734**	**2,73**	**0.012**
Δ*E* Black	Standard length	1.917	1,73	0.17
	Pollution	2.22	2,73	0.116
	**Population(Pollution)**	**6.836**	**2,73**	**0.002**

**TABLE 4:** Pairwise comparisons of estimated marginal means of (A) area of ornamentation and (B) colour intensity (Δ*E*) between pollution regimes and populations from the same pollution regime. Pollution regime names were simplified: AP = Anthropogenic Pollution, NP = Non‐Polluted, PL = Pitch Lake. As the Pitch Lake was considered its own pollution regime it was excluded from pairwise population comparisons. We used an adjusted alpha‐level (Bonferroni: *α* = 0.025) for ascertaining significance when comparing pollution regimes, and significant *p* values are highlighted in bold.

Dependent variable	Comparison	Mean difference	SE	*p*
(A) Area of ornamentation				
(A1) Pollution regime				
PC1 (Orange)	AP vs. NP	0.027	0.17	0.872
	**AP vs. PL**	**−0.725**	**0.223**	**0.001**
	**NP vs. PL**	**−0.753**	**0.216**	**0.001**
PC2 (Iridescence)	**AP vs. NP**	**0.504**	**0.168**	**0.003**
	**AP vs. PL**	**0.841**	**0.22**	**< 0.001**
	NP vs. PL	0.337	0.213	0.116
PC3 (Black)	AP vs. NP	0.08	0.174	0.648
	**AP vs. PL**	**0.707**	**0.229**	**0.002**
	**NP vs. PL**	**0.627**	**0.221**	**0.005**
(A2) Population‐within‐pollution				
PC1 (Orange)	P1 vs. P2	−0.115	0.27	0.671
	N1 vs. N2	−0.156	0.223	0.485
PC2 (Iridescence)	**P1 vs. P2**	0.037	0.267	0.889
	N1 vs. N2	−0.292	0.22	0.186
PC3 (Black)	P1 vs. P2	−0.288	0.277	0.299
	N1 vs. N2	0.359	0.228	0.118
(B) Colour intensity				
(B1) Pollution regime				
Δ*E* Orange	**AP vs. NP**	**3.069**	**1.118**	**0.008**
	AP vs. PL	0.558	1.53	0.716
	NP vs. PL	−2.511	1.575	0.115
Δ*E* Iridescence	AP vs. NP	1.559	1.338	0.248
	**AP vs. PL**	**4.705**	**1.831**	**0.012**
	NP vs. PL	**6.265**	**1.885**	**0.001**
Δ*E* Black	AP vs. NP	0.06	0.696	0.931
	AP vs. PL	−1.87	0.952	0.053
	NP vs. PL	−1.93	0.98	0.053
(B2) Population‐within‐pollution				
Δ*E* Orange	**P1 vs. P2**	**−5.809**	**1.741**	**0.001**
	N1 vs. N2	1.228	1.654	0.46
Δ*E* Iridescence	**P1 vs. P2**	**−5.503**	**2.084**	**0.001**
	N1 vs. N2	2.358	1.979	0.237
Δ*E* Black	**P1 vs. P2**	**−4.001**	**1.084**	**< 0.001**
	N1 vs. N2	−0.323	1.029	0.755

Year only had a significant effect on iridescence (PC2: *p* = 0.014), with no effect on either orange (PC1) or black (PC3). Iridescence strongly increased between 2018 and 2019 (Figure 2B) with a slight increase in orange and no change in black.

**FIGURE 3:**
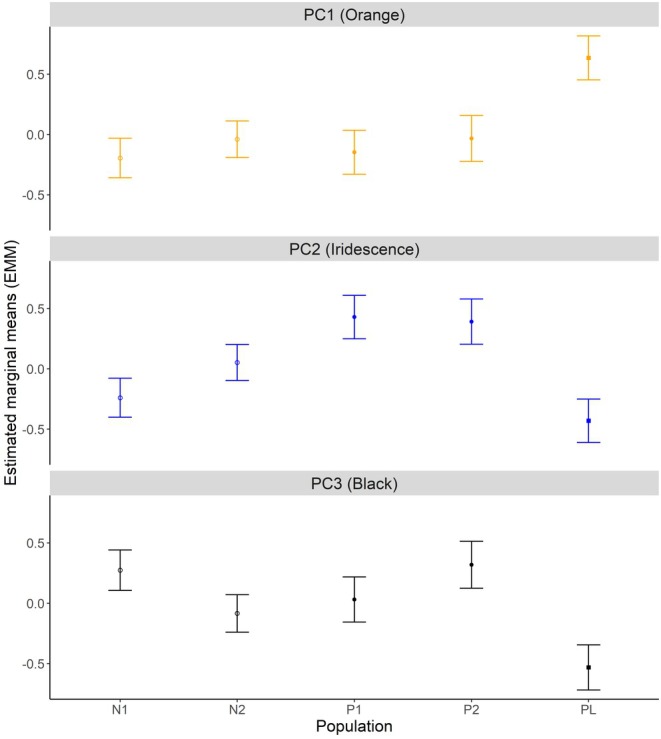
Estimated marginal means (±SE) for area of ornamentation between populations. Pollution regime indicated by shape, with open circle denoting ‘non‐polluted’, closed circle as ‘anthropogenically polluted’ and closed square as ‘Pitch Lake’.

There was a significant interaction effect of ‘year‐by‐pollution’ for orange (PC1: *p* = 0.044) as area of orange increased between 2018 and 2019 in anthropogenically polluted habitats, decreased in the Pitch Lake, and only increased a small amount in non‐polluted habitats. Additionally, there was a non‐significant trend of ‘year‐by‐pollution’ for iridescence (PC1: *p* = 0.067), which was due to the fact that iridescence increased strongly in polluted habitats from 2018 to 2019, increased less strongly in non‐polluted habitats, and did not change in the Pitch Lake. There was no effect of year‐by‐population (pollution) on any of the individual PCs (Figure 3, Table 4).

### 3.2 Colour Intensity

Results of the two MANCOVAs found that the covariate standard length had no influence on colour intensity (*p =* 0.287, Table 2B), but that both pollution regime (*p* = 0.002) and population(pollution) (*p* = 0.007) significantly affected intensities.

Results of post hoc ANCOVAs confirmed that there was no relationship between colour intensity and standard length for any of the three colour types (Table 3B).

**FIGURE 4:**
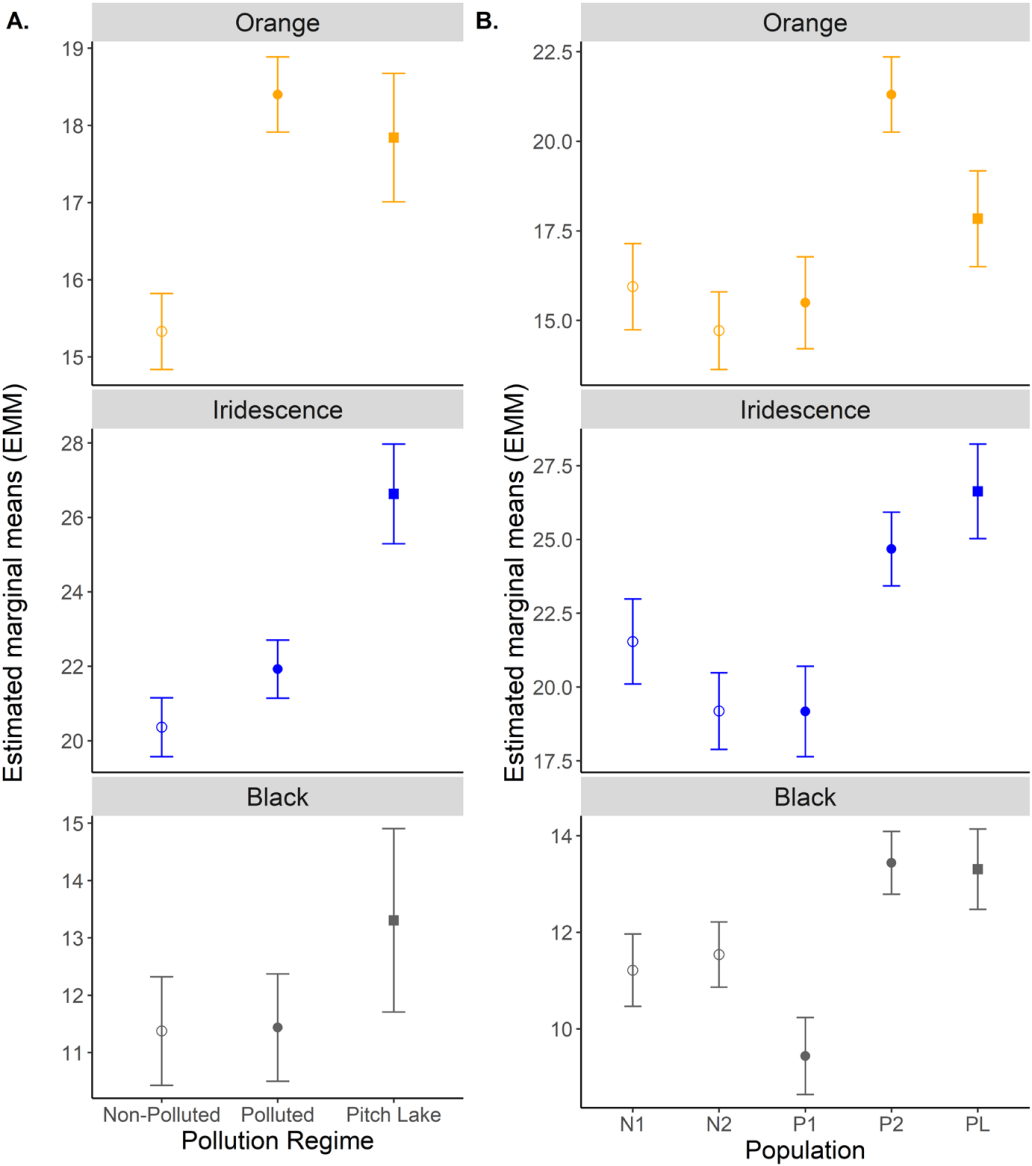
(A) Estimated marginal means (±SE) indicating change in colour intensity (Δ*E*) between pollution regimes and (B) between populations. Pollution regime indicated by shape, with open circle denoting ‘non‐polluted’, closed circle as ‘anthropogenically polluted’ and closed square as ‘Pitch Lake’.

Regarding effects of pollution regime on individual colour, both iridescence (*p =* 0.006) and orange (*p =* 0.024) showed a significant effect, while black did not (*p* = 0.179; Table 3A). Regarding iridescence, post hoc pairwise comparisons found that while polluted and non‐polluted habitats did not differ, the Pitch Lake differed from both, having the highest mean Δ*E* (Figure 4A, Table 4B). For orange intensity, post hoc comparisons revealed that polluted and non‐polluted habitats differed significantly, with Δ*E* being largest in guppies from polluted habitats, but that the Pitch Lake did not differ significantly from either (Table 4B).

All three colour types showed significant variation between populations within pollution regimes (orange: *p =* 0.002, iridescence: *p =* 0.013, black: *p =* 0.002; Table 3B, Figure 4B). This was due to considerable variation between the two anthropogenically polluted populations—in both orange and black colour intensity, population P1 differed significantly from P2 (Table 4B, Figure 4B). There was no significant variation between non‐polluted populations for any of the three colour type intensities.

## 4 Discussion

### 4.1 Crude Oil Pollution

Contrary to our predictions, crude oil pollution did not have a straightforward impact on ornamentation, but in agreement with our third prediction, the Pitch Lake guppies often exhibited a unique response. Whilst there were significant differences in both the area and intensity of ornamentation, differences between pollution regimes were not uniform between ornamentation types. This highlights a lack of uniformity in exposure to oil‐related hydrocarbons, as fish from anthropogenically polluted habitats displayed different phenotypes to those from the naturally polluted Pitch Lake, and even fish from the two anthropogenically polluted habitats were often significantly different from each other.

The fact that crude oil pollution (whether of natural cause or through anthropogenic activities) did not have a direct impact on area of ornamentation is noteworthy. Crude oil has documented negative effects on DNA integrity and liver health (Santos et al. 2024), on fish embryonic development (Incardona et al. 2013) and immune functioning (Bayha et al. 2017), factors that should have a direct impact on ornamentation development. Moreover, detoxification of pollutants is metabolically costly and should reduce energy available for development and maintenance of other traits (Handy et al. 1999; Marchand et al. 2004), such as ornamentation. Additionally, crude oil pollution can reduce species richness (la De Huz et al. 2005; Peterson 2001), which may alter the diet of guppies, potentially impacting also the availability of carotenoid‐rich food. Nonetheless, no overall crude oil‐pollution effects on guppy ornamentation were uncovered. In the subsequent sections we will try to elucidate this pattern further.

### 4.2 Anthropogenic Crude Oil Pollution

Despite the lack of uniform response, there were some differences between anthropogenically polluted and non‐polluted habitats. For numbers of spots and area of ornamentation, guppies from anthropogenically polluted sites had more iridescent ornamentation than guppies from either non‐polluted sites or the Pitch Lake. For intensity, orange Δ*E* was greater in guppies from anthropogenically polluted habitats than in those from non‐polluted waters. This last trend was driven by lower L* values in polluted habitats with very little change in a* and b* (Table S3, Figure S3), meaning a generally darker colour with little difference in hue.

Increased area of iridescence may be driven by a number of environmental and ecological factors linked to anthropogenic oil pollution. One potential consequence of crude oil pollution is an increase in turbidity as crude oil binds with particulate matter (Rügner et al. 2013; Schwientek et al. 2013). Further, crude oil pollution can affect light attenuation in the water (Haule et al. 2015; Król et al. 2006), altering guppy vision and affecting which ornamentation colours are most visible (Endler 1991). An increase in turbidity has been found to lead to an increase in the area of ultra‐violet (UV) ornamentation in male guppies (Camargo‐Dos‐Santos et al. 2021), and in a related poecilid fish, *Gambusia hubbsi*, changes in water colour were associated with an increase in size of iridescent patches (Martin et al. 2014). Although turbidity was not directly measured, anthropogenically polluted sites had higher total dissolved solids (TDS) than non‐polluted sites (Table S1), which may indicate greater suspended particulate and greater turbidity. Analysis of the spectral properties of the water in these polluted habitats may shed further light on these potential factors.

A greater area of iridescence in male guppies is classically associated with predator release: decreased predation pressure leads to increased area of ornamentation, including iridescence (Kodric‐Brown 1985; Endler 1980, 1983). An increase in pollution of various forms is associated with declines in predator richness and/or loss of top predators (Gomes‐Silva, Cyubahiro, et al. 2020; Gomes‐Silva, Pereira, et al. 2020; Kean et al. 2021; Mor et al. 2022), although some predators of at least juvenile guppies were also recorded at P2, one of the anthropogenically polluted sites used here (the killifish, *Anablepsoides hartii*; Table S1). It must also be noted that release from predation pressure is primarily associated with an increase in orange ornamentation (Kodric‐Brown 1985; Endler 1980, 1983) which was not observed in the present study. Other physiological or ecological factors, such as limitations in carotenoid availability, may constrain the development of orange ornamentation despite reduced predation pressure. Further investigation into spatial differences in predation pressure would help to clarify the extent to which predator release contributes to variation in ornamentation.

Whilst the area of orange ornamentation was not affected by anthropogenic oil pollution, orange colour intensity was darker in males from anthropogenically polluted habitats compared to those from non‐polluted ones. However, this difference was driven by population P2, with P1 having similar Δ*E* to the non‐polluted populations. This again highlights the non‐uniform effects of oil pollution. As colour intensity is a phenotypically plastic trait, it is more sensitive to environmental changes that may be population specific.

Differences in diet such as greater detritus reliance seen in guppies exposed to pollution (De Carvalho et al. 2019) may alter dietary carotenoid availability and lead to reduced orange intensity. Whilst gut content analysis on fish from the same system did not detect any differences in diet or foraging opportunities between pollution regime with regards to invertebrate prey (McGovern et al., unpublished), this does not discount differences in unicellular algae consumption. Further, increased oxidative stress (Livingstone 2001; Livingstone et al. 1993) may increase demand for carotenoids in roles other than ornamentation like as an antioxidant or immune system functioning (Houde and Torio 1992; Zhang et al. 2019). We call on further investigation specifically into the differences in carotenoid availability between the diets of guppies from low and high pollution habitats.

Intensity of both iridescence and black ornamentation showed no significant difference between anthropogenically polluted and non‐polluted habitats. However, when comparing L*a*b* values for these colour types, P2 had lower L* values than non‐polluted habitats, indicating a darker colouration (Table S3, Figure S2). Melanin can be produced to counter increased oxidative stress (Hou et al. 2022; Plonka et al. 2009) such as that caused by PAH exposure, and a study of guppies exposed to a chemical pollutant (Triphenyltin) found increased melanin production that distributed diffusely below the skin, creating an appearance of dull, darker colours (Hou et al. 2022). Preliminary investigations into non‐ornamentation colouration suggest fish from polluted habitats may have lower L* values indicating darker body colour (Table S4), which supports the idea of overall increase in melanin production in polluted habitats, and particularly in population P2. However, methodological difficulties in differentiating ‘fuzzy black’ or ‘brown’ ornamentation (Kemp et al. 2008) from non‐ornamentation colour limit our ability to unbiasedly measure diffuse melanin across total body. Further investigation directly measuring melanin pigmentation in female guppies, which lack any colour ornamentation, is necessary to verify these preliminary findings. It is also important to stress that we did not find a significant difference in the intensity of black ornamentation patches, but this does not preclude that melanin increased in other parts of the body.

Ornamentation is a significant factor in guppy mate selection (Endler 1983), and males that can maintain bright colouration despite the deleterious effects of pollution are likely to be at an advantage for attracting female mates. The fact that there was no consistent reduction in area or intensity of ornamentation in anthropogenically polluted habitats (in fact, we detected some increases) suggests these traits are still selected for despite the likely bigger strain on producing these colours. Therefore, selection for ornamentation may be stronger than that for metabolic or developmental adaptations that do not have immediate and direct reproductive benefits, leading to rapid adaptation in ornamentation. Future experimental and developmental studies should investigate this and previous points further.

### 4.3 The Pitch Lake

While we uncovered an overall effect of pollution regime on both area and intensity ornamentation, post hoc comparisons revealed many of these effects were driven by differences between the Pitch Lake versus anthropogenically polluted and non‐polluted habitats. This is of note because the Pitch Lake is the only naturally oil‐polluted habitat in our dataset, and the fish living within are subject to different environmental pressures compared to surrounding sites.

With regards to area of ornamentation, the Pitch Lake differed from both anthropogenically polluted and non‐polluted habitats for both orange and black colouration. Specifically, the number of spots and area of orange ornamentation was greater in the Pitch Lake than in all other populations, regardless of pollution level, while the area and number of black spots were smallest in the Pitch Lake. Orange ornamentation is the product of a combination of carotenoids and drosopterins, with unicellular algae having been identified as the primary source for carotenoids in guppies while drosopterins can be synthesised *de novo* (Grether, Hudon, and Endler 2001). Moreover, Grether, Hudon, and Endler (2001) showed that guppies do not utilise drosopterins to compensate for low carotenoid availability but rather that the two are linked. Thus, the increased orange ornamentation in the Pitch Lake would suggest that despite its toxicity, unicellular algae (or an alternative carotenoid source) must be available in large enough quantities for guppies to have even more body area covered in orange/red than elsewhere. Alternatively, it is possible that Pitch Lake guppies have evolved the ability to increase drosopterin deposition into chromatophores if carotenoids are scarce. This hypothesis would match the fact that Pitch Lake guppies had the lowest L* and greatest a* values of all populations, indicating orange ornamentation was darker and redder (Table S3, Figure S2). Greater red pigmentation could indicate a higher proportion of drosopterin compared to carotenoid pigmentation (Grether, Hudon, and Endler 2001). Future studies will have to investigate the potential presence of unicellular algae and other potential sources of carotenoids in the Pitch Lake further.

Another possible explanation for increased orange ornamentation in the Pitch Lake involves the potential anti‐parasitic properties of the pitch. Schelkle et al. (2012) found that guppies from the Pitch Lake had lower levels of ectoparasite infection, and that exposing infected fish to Pitch Lake water cured them of their ectoparasitic infection. The carotenoids that, together with drosopterins, produce orange colouration (Grether, Hudon, and Endler 2001) are also involved in immune functioning, and parasite infection has been found to immediately reduce the chroma of orange ornamentation in guppies (Houde and Torio 1992; Kolluru et al. 2006; Stephenson et al. 2020). Given these apparent anti‐parasitic properties of the Pitch Lake waters, this may reduce the constraints on the trade‐off for carotenoid allocation, allowing fish to develop a greater number of spots and a larger area of orange ornamentation even if there is no difference in the availability of carotenoids (or algae) between the Pitch Lake and the other polluted habitats. However, it is important to note that Schelkle et al. (2012) only focused on ectoparasitic *Gyrodactylus* infections, and so we currently do not know if other parasites are similarly affected.

With respect to the low amount of black body ornamentation in the Pitch Lake, this is the opposite pattern to orange. This may be due to the aforementioned parasite release and increased orange ornamentation. Increased orange means physically there is less space on the body for other colours. Orange is also a more ‘honest’ sexual signal as it is produced by the diet, so females may show preference for this (Grether et al. 1999), reducing selection for black and iridescence. Additionally, the area of black colouration has been found to reduce as canopy cover decreases (Millar et al. 2006). The Pitch Lake has almost no canopy cover, with only a handful of shrubs and small trees surrounding the periphery. Potentially a number of these factors have led to the unique phenotype in the Pitch Lake that was not found in anthropogenically polluted habitats.

The Pitch Lake also differed from both other habitat types in terms of intensity of iridescence by sporting the greatest Δ*E* (Figure 4B) and lowest L* values (Figure S2) of all populations. Whilst we hypothesised that anthropogenically polluted habitats may act as predator refuge due to the lack of observed predator species and the increased iridescence ornamentation, the opposite has been found in the Pitch Lake. A few piscivorous fishes have been reported from the Pitch Lake [ *A. hartii* and the Guyana leaffish ( *Polycentrus schomburgkii* ); Mohammed et al. 2010; Santi et al. 2021; Table S1] and several piscivorous birds frequent the Pitch Lake in large numbers (including Black Skimmers, *Rhyncops niger*; Yellow‐Billed Terns, *Sternula superciliaris*; and Snowy Egrets, ( *Egretta thula* ); Santi et al. 2019, 2021; H. McGovern, personal observation). Variation in iridescence may be dependent on the taxonomic identity of the predator (Millar et al. 2006). Whilst the impact of avian predation on guppy colouration has not been directly tested, avian vision is sensitive to UV wavelengths (Håstad et al. 2005) and therefore may select against iridescent colouration in guppies. Nonetheless, the overall community structure of the Pitch Lake is not well known and therefore it is not possible to draw substantial conclusions about fish predation impacts on ornamentation at this point.

The above patterns warrant an explanation as to why the Pitch Lake guppies are so different from guppies in anthropogenically polluted waters. The Pitch Lake was formed in the late Miocene through an upwelling of bitumen and oil (Ponnamperuma and Pering 1967), meaning these ‘pollutant’ compounds have long been established in the local environment, compared to the much more recent anthropogenic pollution found at the other polluted sites (where pollution is likely to be at the most a couple of decades old, Rolshausen et al. 2015; Santi et al. 2021). This could result in important differences between the Pitch Lake and the anthropogenically polluted habitats. First, the oil and bitumen in the Pitch Lake are mostly situated at the bottom of the water bodies, where they have long settled, and are covered by a layer of sediment and detritus (H. McGovern, personal observation). This could reduce the amount of leakage of toxic compounds from the crude oil and bitumen, and our own water samples collected in 2024 seem to confirm this (McGovern et al., unpublished data). In contrast, in the anthropogenically polluted sites, crude oil usually enters the environments from the side, and thus, is visible as oil slick on the surface of the water (H. McGovern, personal observation). This is likely to result in a vastly different amount of leakage of toxic compounds into the water column, which again, is confirmed by our 2024 water samples (McGovern et al., unpublished data). Second, even though it is not known when guppies colonised the Pitch Lake, population genetic analysis identified Pitch Lake guppies as genetically unique (Willing et al. 2010; McGovern et al., unpublished data), suggesting this population has been reproductively isolated for many (i.e., evolutionarily significant) generations. Reproductive isolation and strong selection pressures in the form of crude oil exposure, along with potential historic bottlenecks and genetic drift, could have led to genetic distinctiveness. This is turn could manifest itself via distinct ornamentation patterns compared to those exhibited by other guppies in the region.

### 4.4 Temporal and Population‐Level Variation

Area of iridescent ornamentation changed between 2018 and 2019, driven by a strong increase in the number of spots and area of iridescence in anthropogenically polluted habitats, a less pronounced increase in non‐polluted waters, and the absence of change in the Pitch Lake. Consistent changes across all populations suggest region‐wide environmental variation is driving this annual increase. One possible environmental driver is temperature: experimental studies have found area of iridescence (Rahman et al. 2020) increased towards an optimal temperature of 28°C. Average annual air temperature in the region increased between years from 26.84°C to 27.16°C, which is closer to this optimum (World Bank 2024). This hypothesis would also explain the lack of increase in iridescence in the Pitch Lake population, as the Pitch Lake has water temperatures of up to 32°C (Santi et al. 2019; Schelkle et al. 2012) and therefore already well exceeds this thermal optimum.

A similar pattern was found in orange ornamentation, as area increased greatly in anthropogenically polluted habitats and marginally increased in non‐polluted habitats, but decreased in the Pitch Lake. This large change in a genetically determined trait indicates a severe change in selection pressure between years, particularly within polluted habitats. This could be due to several factors including the aforementioned temperature increase, which has been found to increase orange chroma (Breckels and Neff 2013), though no multigenerational temperature studies have confirmed effects on orange area specifically. However, we currently lack enough information on year‐specific changes in ecological variables in the studied populations to be able to properly speculate on what might have caused these inconsistent patterns across populations.

The fact that the area of iridescence and orange increased more strongly between years in anthropogenically polluted habitats may be due to the high stress, unpredictable environment leading to greater phenotypic and genetic variation. Stress can increase genetic variation through increased mutation and recombination rates (Hoffmann and Hercus 2000), and indeed, PAHs have been found to be mutagenic (Samanta et al. 2002; Santos et al. 2024) which may lead to greater genetic variation in these affected populations. Recent fieldwork in the region in 2024 and 2025 has revealed that the concentration of oil pollution seems to fluctuate temporally within already‐polluted habitats (H. McGovern, personal observation). These acute spills can temporarily increase the concentration of environmental crude oil well above the norm, putting greater strain on the guppy populations and physiological traits like ornamentation. In addition, stress can select for greater phenotypic plasticity, particularly in environments where the intensity of the stressor can vary within an organism's lifetime (Gabriel 2005). High stress, volatile environments select against directional selection and towards flexibility, which may explain the larger change in ornamentation seen in these high pollution populations.

Colour intensity varied significantly between populations of the same habitat type for all three colour types, but this was essentially driven only by differences observed between the two anthropogenically polluted habitats (P1 and P2). This, combined with the greater annual variation in area of ornamentation, indicates the non‐uniform and variable responses to crude oil exposure across both time and space. This may again be driven by variability of crude oil pollution: recent acute spills may increase stress beyond usual physiological tolerances, leading to plastic responses like a reduction in colour intensity. Whilst unfortunately there is no available data for location or timing of acute spills in the region, it was noted during fieldwork that P2 is located less than 500 m from a pumpjack drill, making it more at risk of acute spills. P2 also had the highest conductivity and TDS of all sites (Table S1). This could explain why P2 had greater Δ*E* and, therefore, darker colouration as fish may have recently been exposed to an acute spill. However, further data is required on location and size of acute spills to investigate this relationship further.

## 5 Conclusions

We found no consistent pattern of male ornamentation in oil‐polluted habitats across populations and years, despite the detrimental environmental and physical effects of oil pollution. This is further evidence (see also Santi et al. 2019, 2021) that the impacts of oil pollution are not uniform for all habitats and may vary through interactions with additional environmental factors. Thus, extreme habitats derived from oil pollution are different from other types of extreme freshwater habitats, such as toxic sulphide springs (reviewed in Tobler et al. 2018) or subterranean habitats (reviewed in Niemiller and Soares 2015), which often result in strongly convergent phenotypes for the inhabiting organisms.

This is particularly true for the Pitch Lake population, which was markedly different in area of orange and black, and intensity of iridescence ornamentation (this study) as well as life histories and body shape (Santi et al. 2021) compared to all other oil‐polluted populations in this region. We speculate that the unique ecological and environmental aspects of this habitat including high temperature and low pH (Santi et al. 2019), high density of predators (Santi et al. 2019, 2021), and potentially low levels of parasitism (Schelkle et al. 2012), coupled with genetic distinctiveness (Willing et al. 2010) and possible bottlenecking, have led to unique phenotypes within this population.

This emphasises a need to consider pollution impacts within a larger framework of ecological and evolutionary variability (i.e., adaptive potential of the focal organism). Only through a more integrated approach will we be able to accurately predict responses to, and potentially mitigate the negative effects of, anthropogenic oil pollution. We therefore call for more research on diverse oil‐polluted environments across the globe to better identify the shared and unique aspects of this widespread form of pollution on aquatic freshwater systems.

## Author Contributions

**Hannah Rose McGovern:** data curation (lead), formal analysis (equal), funding acquisition (equal), methodology (equal), validation (equal), visualization (lead), writing – original draft (lead), writing – review and editing (equal). **Francesco Santi:** conceptualization (equal), investigation (lead), methodology (equal), writing – review and editing (equal). **Amy Deacon:** resources (equal), writing – review and editing (equal). **Rüdiger Riesch:** conceptualization (equal), funding acquisition (equal), methodology (equal), project administration (lead), resources (equal), supervision (lead), writing – review and editing (equal).

## Funding

The research presented in this article was supported by the Natural Environment Research Council grant number NE/S007229/1 as a Studentship awarded to Hannah Rose McGovern, funded from 2022 to 2026. Additional funding was awarded by the Royal Holloway Doctoral School and by the Department of Biological Sciences at Royal Holloway University of London.

## Ethics Statement

For the collection of these data, we have adhered to the Guidelines for the Use of Animals in Research. The study reported here is in agreement with the respective laws in Trinidad and Tobago and the United Kingdom. The sampling protocol was approved by the Royal Holloway Animal Welfare and Ethical Review Body (No. RHUL‐NRR‐0003‐2017).

## Conflicts of Interest

The authors declare no conflicts of interest.

## Supporting information


**Appendix S1:** ece373105‐sup‐0001‐AppendixS1.docx.

## Data Availability

The dataset associated with this work is available via Figshare (https://figshare.com/s/b2f91eef6a93800e6c16).

## References

[ece373105-bib-0001] Altman, D. G. 1999. Practical Statistics for Medical Research. Chapman & Hall/CRC Press.

[ece373105-bib-0002] Araújo, F. G. , B. Pinto , and T. P. Teixeira . 2009. “Distribution of Guppies *Poecilia reticulata* (Peters, 1860) and *Phalloceros caudimaculatus* (Hensel, 1868) Along a Polluted Stretch of the Paraíba Do Sul River, Brazil.” Brazilian Journal of Biology 69: 41–48. 10.1590/s1519-69842009000100005 19347144

[ece373105-bib-0003] Arellano‐Aguilar, O. , and C. M. Garcia . 2008. “Exposure to Pesticides Impairs the Expression of Fish Ornaments Reducing the Availability of Attractive Males.” Proceedings of the Royal Society B: Biological Sciences 275: 1343–1351. 10.1098/rspb.2008.0163 PMC2602681 18348963

[ece373105-bib-0004] Bayha, K. M. , N. Ortell , C. N. Ryan , et al. 2017. “Crude Oil Impairs Immune Function and Increases Susceptibility to Pathogenic Bacteria in Southern Flounder.” PLoS One 12: e0176559. 28464028 10.1371/journal.pone.0176559 PMC5413019

[ece373105-bib-0005] Bray, J. , and S. Maxwell . 1985. Multivariate Analysis of Variance (Quantitative Applications in the Social Sciences 54). SAGE.

[ece373105-bib-0006] Breckels, R. D. , and B. D. Neff . 2013. “The Effects of Elevated Temperature on the Sexual Traits, Immunology and Survivorship of a Tropical Ectotherm.” Journal of Experimental Biology 216: 2658–2664. 23531818 10.1242/jeb.084962

[ece373105-bib-0007] Camargo‐Dos‐Santos, B. , B. B. Gonçalves , M. S. Bellot , I. I. Guermandi , B. Assaf , and P. C. Giaquinto . 2021. “Water Turbidity‐Induced Alterations in Coloration and Courtship Behavior of Male Guppies (*Poecilia reticulata*).” Acta Ethologica 24: 127–136.

[ece373105-bib-0008] Cattelan, S. , J. P. Evans , F. Garcia‐Gonzalez , E. Morbiato , and A. Pilastro . 2020. “Dietary Stress Increases the Total Opportunity for Sexual Selection and Modifies Selection on Condition‐Dependent Traits.” Ecology Letters 23: 447–456. 31840374 10.1111/ele.13443

[ece373105-bib-0009] Darwall, W. , V. Bremerich , A. de Wever , et al. 2018. “The Alliance for Freshwater Life: A Global Call to Unite Efforts for Freshwater Biodiversity Science and Conservation.” Aquatic Conservation: Marine and Freshwater Ecosystems 28: 1015–1022.

[ece373105-bib-0010] De Carvalho, D. R. , A. S. Flecker , C. B. Mascarenhas Alves , J. P. Sparks , and P. Santos Pompeu . 2019. “Trophic Responses to Aquatic Pollution of Native and Exotic Livebearer Fishes.” Science of the Total Environment 681: 503–515. 31128341 10.1016/j.scitotenv.2019.05.092

[ece373105-bib-0011] Devigili, A. , J. P. Evans , A. Di Nisio , and A. Pilastro . 2015. “Multivariate Selection Drives Concordant Patterns of Pre‐ and Postcopulatory Sexual Selection in a Livebearing Fish.” Nature Communications 6: 8291. 10.1038/ncomms9291 PMC4579849 26369735

[ece373105-bib-0012] Dudgeon, D. , A. H. Arthington , M. O. Gessner , et al. 2006. “Freshwater Biodiversity: Importance, Threats, Status and Conservation Challenges.” Biological Reviews of the Cambridge Philosophical Society 81: 163–182. 16336747 10.1017/S1464793105006950

[ece373105-bib-0013] Endler, J. A. 1980. “Natural Selection on Color Patterns in *Poecilia reticulata* .” Evolution 34: 76–91. 28563214 10.1111/j.1558-5646.1980.tb04790.x

[ece373105-bib-0014] Endler, J. A. 1983. “Natural and Sexual Selection on Color Patterns in Poeciliid Fishes.” Environmental Biology of Fishes 9: 173–190.

[ece373105-bib-0015] Endler, J. A. 1991. “Variation in the Appearance of Guppy Color Patterns to Guppies and Their Predators Under Different Visual Conditions.” Vision Research 31: 587–608. 1843763 10.1016/0042-6989(91)90109-i

[ece373105-bib-0016] Fernandes, I. M. , Y. F. Bastos , D. S. Barreto , L. S. Lourenço , and J. M. Penha . 2017. “The Efficacy of Clove Oil as an Anaesthetic and in Euthanasia Procedure for Small‐Sized Tropical Fishes.” Brazilian Journal of Biology 77: 444–450. 10.1590/1519-6984.15015 27683808

[ece373105-bib-0017] Freedman, B. 1995. Environmental Ecology: The Ecological Effects of Pollution, Disturbance, and Other Stresses (Illustrated, Ed.). Academic Press.

[ece373105-bib-0018] Gabriel, W. 2005. “How Stress Selects for Reversible Phenotypic Plasticity.” Journal of Evolutionary Biology 18: 873–883. 16033559 10.1111/j.1420-9101.2005.00959.x

[ece373105-bib-0019] Godin, J.‐G. J. , and H. E. McDonough . 2003. “Predator Preference for Brightly Colored Males in the Guppy: A Viability Cost for a Sexually Selected Trait.” Behavioral Ecology 14: 194–200.

[ece373105-bib-0020] Gomes‐Silva, G. , E. Cyubahiro , T. Wronski , R. Riesch , A. Apio , and M. Plath . 2020. “Water Pollution Affects Fish Community Structure and Alters Evolutionary Trajectories of Invasive Guppies ( *Poecilia reticulata* ).” Science of the Total Environment 730: 138912. 32402962 10.1016/j.scitotenv.2020.138912

[ece373105-bib-0021] Gomes‐Silva, G. , B. B. Pereira , K. Liu , et al. 2020. “Using Native and Invasive Livebearing Fishes (Poeciliidae, Teleostei) for the Integrated Biological Assessment of Pollution in Urban Streams.” Science of the Total Environment 698: 134336. 31783440 10.1016/j.scitotenv.2019.134336

[ece373105-bib-0022] Government of the Republic of Trinidad and Tobago . 2024. Oil and Gas Industry Overview. Ministry of Energy and Energy Industries. https://www.energy.gov.tt/our‐business/oil‐and‐gas‐industry/.

[ece373105-bib-0023] Grether, G. F. , J. Hudon , and J. A. Endler . 2001. “Carotenoid Scarcity, Synthetic Pteridine Pigments and the Evolution of Sexual Coloration in Guppies ( *Poecilia reticulata* ).” Proceedings of the Royal Society of London. Series B: Biological Sciences 268: 1245–1253. 10.1098/rspb.2001.1624 PMC1088733 11410150

[ece373105-bib-0024] Grether, G. F. , J. Hudon , and D. F. Millie . 1999. “Carotenoid Limitation of Sexual Coloration Along an Environmental Gradient in Guppies.” Proceedings of the Royal Society of London. Series B: Biological Sciences 266: 1317–1322.

[ece373105-bib-0025] Grether, G. F. , D. F. Millie , M. J. Bryant , D. N. Reznick , and W. Mayea . 2001. “Rain Forest Canopy Cover, Resource Availability, and Life History Evolution in Guppies.” Ecology 82: 1546–1559.

[ece373105-bib-0078] Handy, R. D. , D. W. Sims , A. Giles , H. A. Campbell , and M. M. Musonda . 1999. “Metabolic Trade‐Off Between Locomotion and Detoxification for Maintenance of Blood Chemistry and Growth Parameters by Rainbow Trout (*Oncorhynchus mykiss*) During Chronic Dietary Exposure to Copper.” Aquatic Toxicology 47: 23–41.

[ece373105-bib-0082] Håstad, O. , E. Ernstdotter , and A. Ödeen . 2005. “Ultraviolet Vision and Foraging in Dip and Plunge Diving Birds.” Biology Letters 1: 306–309. 17148194 10.1098/rsbl.2005.0320 PMC1617148

[ece373105-bib-0027] Haule, K. , M. Darecki , and H. Toczek . 2015. “Light Penetration in Seawater Polluted by Dispersed Oil: Results of Radiative Transfer Modelling.” Journal of the European Optical Society‐Rapid Publications 10: 15052.

[ece373105-bib-0028] Hoffmann, A. A. , and M. J. Hercus . 2000. “Environmental Stress as an Evolutionary Force.” Bioscience 50: 217–226.

[ece373105-bib-0029] Hou, Y. , L. Wang , Y. Jin , et al. 2022. “Triphenyltin Exposure Induced Abnormal Morphological Colouration in Adult Male Guppies ( *Poecilia reticulata* ).” Ecotoxicology and Environmental Safety 242: 113912. 35905627 10.1016/j.ecoenv.2022.113912

[ece373105-bib-0030] Houde, A. E. 1992. “Sex‐Linked Heritability of a Sexually Selected Character in a Natural Population of *Poecilia reticulata* (Pisces: Poeciliidae) (Guppies).” Heredity 69: 229–235.

[ece373105-bib-0031] Houde, A. E. 1997. “Evolutionary Mismatch of Mating Preferences and Male Colour Patterns in Guppies.” Animal Behaviour 53: 343–351.

[ece373105-bib-0032] Houde, A. E. , and A. J. Torio . 1992. “Effect of Parasitic Infection on Male Color Pattern and Female Choice in Guppies.” Behavioral Ecology 3: 346–351.

[ece373105-bib-0034] Incardona, J. P. , T. L. Swarts , R. C. Edmunds , et al. 2013. “Exxon Valdez to Deepwater Horizon: Comparable Toxicity of Both Crude Oils to Fish Early Life Stages.” Aquatic Toxicology 142–143: 303–316. 10.1016/j.aquatox.2013.08.011 24080042

[ece373105-bib-0035] Kean, E. F. , R. F. Shore , G. Scholey , R. Strachan , and E. A. Chadwick . 2021. “Persistent Pollutants Exceed Toxic Thresholds in a Freshwater Top Predator Decades After Legislative Control.” Environmental Pollution 272: 116415. 33421660 10.1016/j.envpol.2020.116415

[ece373105-bib-0036] Kelly, E. N. , D. W. Schindler , P. V. Hodson , J. W. Short , R. Radmanovich , and C. C. Nielsen . 2010. “Oil Sands Development Contributes Elements Toxic at Low Concentrations to the Athabasca River and Its Tributaries.” Proceedings of the National Academy of Sciences of the United States of America 107: 16178–16183. 20805486 10.1073/pnas.1008754107 PMC2941314

[ece373105-bib-0037] Kemp, D. J. , D. N. Reznick , and G. F. Grether . 2008. “Ornamental Evolution in Trinidadian Guppies ( *Poecilia reticulata* ): Insights From Sensory Processing‐Based Analyses of Entire Colour Patterns.” Biological Journal of the Linnean Society 95: 734–747.

[ece373105-bib-0038] Kodric‐Brown, A. 1985. “Female Preference and Sexual Selection for Male Coloration in the Guppy ( *Poecilia reticulata* ).” Behavioral Ecology and Sociobiology 17: 199–205.

[ece373105-bib-0080] Kolluru, G. R. , G. F. Grether , S. H. South , et al. 2006. “The Effects of Carotenoid and Food Availability on Resistance to a Naturally Occurring Parasite (*Gyrodactylus turnbulli*) in Guppies (*Poecilia reticulata*).” Biological Journal of the Linnean Society 89: 301–309.

[ece373105-bib-0039] Koo, T. K. , and M. Y. Li . 2016. “A Guideline of Selecting and Reporting Intraclass Correlation Coefficients for Reliability Research.” Journal of Chiropractic Medicine 15: 155–163. 27330520 10.1016/j.jcm.2016.02.012 PMC4913118

[ece373105-bib-0040] Kottler, V. A. , I. Koch , M. Flötenmeyer , H. Hashimoto , D. Weigel , and C. Dreyer . 2014. “Multiple Pigment Cell Types Contribute to the Black, Blue, and Orange Ornaments of Male Guppies (*Poecilia reticulata*).” PLoS One 91: e85647. 10.1371/journal.pone.0085647 PMC3899072 24465632

[ece373105-bib-0041] Król, T. , A. Stelmaszewski , and F. Wlodzimierz . 2006. “Variability in the Optical Properties of a Crude Oil—Seawater Emulsion.” Oceanologia 48: 203–211.

[ece373105-bib-0042] la De Huz, R. , M. Lastra , C. Junoy , C. Castellanos , and J. M. Viéitez . 2005. “Biological Impacts of Oil Pollution and Cleaning in the Intertidal Zone of Exposed Sandy Beaches: Preliminary Study of the ‘Prestige’ Oil Spill.” Estuarine, Coastal and Shelf Science 65: 19–29.

[ece373105-bib-0043] Livingstone, D. R. 2001. “Contaminant‐Stimulated Reactive Oxygen Species Production and Oxidative Damage in Aquatic Organisms.” Marine Pollution Bulletin 42: 656–666. 11525283 10.1016/s0025-326x(01)00060-1

[ece373105-bib-0044] Livingstone, D. R. , P. Lemaire , A. Matthews , L. Peters , D. Bucke , and R. J. Law . 1993. “Pro‐Oxidant, Antioxidant and 7‐Ethoxyresorufin O‐Deethylase (EROD) Activity Responses in Liver of Dab ( *Limanda limanda* ) Exposed to Sediment Contaminated With Hydrocarbons and Other Chemicals.” Marine Pollution Bulletin 26: 602–606.

[ece373105-bib-0079] Marchand, J. , L. Quiniou , R. Riso , M. T. Thebaut , and J. Laroche . 2004. “Physiological Cost of Tolerance to Toxicants in the European Flounder *Platichthys flesus*, Along the French Atlantic Coast.” Aquatic Toxicology 70: 327–343. 15588643 10.1016/j.aquatox.2004.10.001

[ece373105-bib-0045] Martin, R. , R. Riesch , J. L. Heinen‐Kay , and R. B. Langerhans . 2014. “Evolution of Male Coloration During a Post‐Pleistocene Radiation of Bahamas Mosquitofish (*Gambusia hubbsi*).” Evolution 68: 397–411. 24111641 10.1111/evo.12277

[ece373105-bib-0046] Millar, N. , D. Reznick , M. Kinnison , and A. Hendry . 2006. “Disentangling the Selective Factors That Act on Male Colour in Wild Guppies.” Oikos 113: 1–12.

[ece373105-bib-0047] Mohammed, R. S. , M. J. McMullan , B. Schelkle , and C. van Oosterhout . 2010. “Colour Variation of an Individual of Hart's Rivulus (*Rivulus hartii*) Found in a Habitat Rich in Polycyclic Aromatic Hydrocarbons in the Pitch Lake of Trinidad.” Ecologia Balkanica 2: 61–63.

[ece373105-bib-0048] Mokrzycki, W. , and M. Tatol . 2011. “Color Difference Delta E—A Survey.” Machine Graphics and Vision 20: 383–411.

[ece373105-bib-0049] Mor, J. R. , I. Muñoz , S. Sabater , L. Zamora , and A. Ruhi . 2022. “Energy Limitation or Sensitive Predators? Trophic and Non‐Trophic Impacts of Wastewater Pollution on Stream Food Webs.” Ecology 103: e03587. 34792187 10.1002/ecy.3587

[ece373105-bib-0050] Niemiller, M. L. , and D. Soares . 2015. “Cave Environments.” In Extremophile Fishes—Ecology, Evolution, and Physiology of Teleosts in Extreme Environments, edited by R. Riesch , M. Tobler , and M. Plath , 161–191. Springer.

[ece373105-bib-0051] Olsgard, F. , and J. Gray . 1995. “A Comprehensive Analysis of the Effects of Offshore Oil and Gas Exploration and Production on the Benthic Communities of the Norwegian Continental Shelf.” Marine Ecology Progress Series 122: 277–306.

[ece373105-bib-0052] Pérez, C. , M. Lores , and A. Velando . 2010. “Oil Pollution Increases Plasma Antioxidants but Reduces Coloration in a Seabird.” Oecologia 163: 875–884. 20532916 10.1007/s00442-010-1677-2

[ece373105-bib-0053] Peterson, C. 2001. “The ‘Exxon Valdez’ Oil Spill in Alaska: Acute, Indirect and Chronic Effects on the Ecosystem.” Advances in Marine Biology 39: 1–103.

[ece373105-bib-0054] Plonka, P. M. , T. Passeron , M. Brenner , et al. 2009. “What Are Melanocytes Really Doing All Day Long?” Experimental Dermatology 18: 799–819. 19659579 10.1111/j.1600-0625.2009.00912.x PMC2792575

[ece373105-bib-0055] Ponnamperuma, C. , and K. L. Pering . 1967. “Aliphatic and Alicyclic Hydrocarbons Isolated From Trinidad Lake Asphalt.” Geochimica et Cosmochimica Acta 31: 1350–1354.

[ece373105-bib-0056] R Core Team . 2024. R: A Language and Environment for Statistical Computing. R Foundation for Statistical Computing. https://www.R‐project.org/.

[ece373105-bib-0057] Rahman, M. M. , I. A. Pinkey , J. Ferthous , et al. 2020. “Modulation of Phenotypic Traits Under Different Rearing Temperatures: Experimental Evidence in Male Guppy (*Poecilia reticulata*).” International Journal of Aquatic Biology 8: 344–364.

[ece373105-bib-0058] Rolshausen, G. , D. A. T. Phillip , D. M. Beckles , et al. 2015. “Do Stressful Conditions Make Adaptation Difficult? Guppies in the Oil‐Polluted Environments of Southern Trinidad.” Evolutionary Applications 8: 854–870. 26495039 10.1111/eva.12289 PMC4610383

[ece373105-bib-0059] Rowe, D. W. , J. B. Sprague , and T. A. Heming . 1983. “Sublethal Effects of Treated Liquid Effluent From a Petroleum Refinery. I. Chronic Toxicity to Flagfish.” Aquatic Toxicology 3: 149–159.

[ece373105-bib-0060] Ruell, E. W. , C. A. Handelsman , C. L. Hawkins , H. R. Sofaer , C. K. Ghalambor , and L. Angeloni . 2013. “Fear, Food and Sexual Ornamentation: Plasticity of Colour Development in Trinidadian Guppies.” Proceedings of the Royal Society B: Biological Sciences 280: 20122019. 10.1098/rspb.2012.2019 PMC3619452 23466982

[ece373105-bib-0061] Rügner, H. , M. Schwientek , B. Beckingham , B. Kuch , and P. Grathwohl . 2013. “Turbidity as a Proxy for Total Suspended Solids (TSS) and Particle Facilitated Pollutant Transport in Catchments.” Environmental Earth Sciences 69: 373–380.

[ece373105-bib-0062] Samanta, S. K. , O. V. Singh , and R. K. Jain . 2002. “Polycyclic Aromatic Hydrocarbons: Environmental Pollution and Bioremediation.” Trends in Biotechnology 20: 243–248. 12007492 10.1016/s0167-7799(02)01943-1

[ece373105-bib-0063] Santi, F. , D. Bierbach , M. Schartl , and R. Riesch . 2019. “Life Histories of Guppies (*Poecilia reticulata* Peters, 1869; Poeciliidae) From the Pitch Lake in Trinidad.” Caribbean Journal of Science 49: 255–262.

[ece373105-bib-0064] Santi, F. , E. Vella , K. Jeffress , A. Deacon , and R. Riesch . 2021. “Phenotypic Responses to Oil Pollution in a Poeciliid Fish.” Environmental Pollution 290: 118023. 34461415 10.1016/j.envpol.2021.118023

[ece373105-bib-0065] Santos, R. M. , A. C. Petry , V. L. Sousa , et al. 2024. “Acute and Subchronic Effects of Petroleum on the Freshwater Fish *Hoplias* Add. *Malabaricus* .” Brazilian Journal of Biology 84: e253731. 10.1590/1519-6984.253731 35019101

[ece373105-bib-0066] Schelkle, B. , R. S. Mohammed , M. P. Coogan , et al. 2012. “Parasites Pitched Against Nature: Pitch Lake Water Protects Guppies ( *Poecilia reticulata* ) From Microbial and Gyrodactylid Infections.” Parasitology 139: 1772–1779. 22831751 10.1017/S0031182012001059

[ece373105-bib-0067] Schneider, C. A. , W. S. Rasband , and K. W. Eliceiri . 2012. “NIH Image to ImageJ: 25 Years of Image Analysis.” Nature Methods 9: 671–675. 22930834 10.1038/nmeth.2089 PMC5554542

[ece373105-bib-0068] Schwientek, M. , H. Rügner , B. Beckingham , B. Kuch , and P. Gratwohl . 2013. “Integrated Monitoring of Particle Associated Transport of PAHs in Contrasting Catchments.” Environmnetal Pollution 172: 155–162. 10.1016/j.envpol.2012.09.004 23063990

[ece373105-bib-0081] Stephenson, J. F. , M. Stevens , J. Troscianko , and J. Jokela . 2020. “The Size, Symmetry, and Color Saturation of a Male Guppy's Ornaments Forecast His Resistance to Parasites.” American Naturalist 196: 597–608. 10.1086/711033 33064581

[ece373105-bib-0069] Sumpter, J. P. 2009. “Protecting Aquatic Organisms From Chemicals: The Harsh Realities.” Philosophical Transactions of the Royal Society A: Mathematical, Physical and Engineering Sciences 367: 3877–3894. 10.1098/rsta.2009.0106 19736226

[ece373105-bib-0070] Tobler, M. , J. L. Kelley , M. Plath , and R. Riesch . 2018. “Extreme Environments and the Origins of Biodiversity: Adaptation and Speciation in Sulphide Springs Fishes.” Molecular Ecology 27: 843–859. 29368386 10.1111/mec.14497

[ece373105-bib-0071] Troscianko, J. , and M. Stevens . 2015. “Image Calibration and Analysis Toolbox—A Free Software Suite for Objectively Measuring Reflectance, Colour and Pattern.” Methods in Ecology and Evolution 6: 1320–1331. 27076902 10.1111/2041-210X.12439 PMC4791150

[ece373105-bib-0072] Wake, H. 2005. “Oil Refineries: A Review of Their Ecological Impacts on the Aquatic Environment.” Estuarine, Coastal and Shelf Science 62: 131–140.

[ece373105-bib-0073] Wickham, H. 2016. Ggplot2: Elegant Graphics for Data Analysis. 2nd ed. Springer International Publishing.

[ece373105-bib-0074] Willing, E. M. , P. Bentzen , C. van Oosterhout , et al. 2010. “Genome‐Wide Single Nucleotide Polymorphisms Reveal Population History and Adaptive Divergence in Wild Guppies.” Molecular Ecology 19: 968–984. 20149094 10.1111/j.1365-294X.2010.04528.x

[ece373105-bib-0075] Winge, Ø. , and E. Ditlevsen . 1947. “Colour Inheritance and Sex Determination in *Lebistes* .” Heredity 1: 65–83.

[ece373105-bib-0076] World Bank . 2024. Climate Change Knowledge Portal. https://climateknowledgeportal.worldbank.org/.

[ece373105-bib-0077] Zhang, J. L. , C. N. Zhang , E. C. Li , et al. 2019. “Triphenyltin Exposure Affects Mating Behaviors and Attractiveness to Females During Mating in Male Guppies ( *Poecilia reticulata* ).” Ecotoxicology and Environmental Safety 169: 76–84. 30423510 10.1016/j.ecoenv.2018.11.011

