# Supplementary material for: Oil Things Bright and Beautiful? How Hydrocarbon Pollution Impacts Guppy Ornamentation
Source: Ecol Evol. 2026 Feb 18;16(2):e73105. doi: 10.1002/ece3.73105 (PMC12916261; doi:10.1002/ece3.73105)
Supplement: Supplementary file 1 — Appendix S1: ece373105‐sup‐0001‐AppendixS1.docx. [file ECE3-16-e73105-s001.docx]

## **Appendix Table 1:** Information on study sites and the numbers of males sampled each year. Water quality factors were measured using a Hach Rugged Field Kit (Hach, Loveland, Colorado, USA) in 2018. Other fish species observed at the study sites are also listed.

| **Name** | **Pollution regime** | **Males sampled** | | **Latitude** | **Longitude** | **Temperature (°C)** | **Dissolved oxygen (mg/L)** | **Specific conductance (uS/cm)** | **Conductivity (uS/cm)** | **Total dissolved solids (mg/L)** | **Salinity (ppt)** | **pH** | **Other fish species** |
| --- | --- | --- | --- | --- | --- | --- | --- | --- | --- | --- | --- | --- | --- |
|  |  | **2018** | **2019** |  |  |  |  |  |  |  |  |  |  |
| PL | Pitch Lake | 13 | 18 | 10.23471 | -61.62496 | 27.7 | 3.29 | 1660 | 1739 | 1079 | 0.83 | 6.69 | *Anablepsoides hartii, Polycentrus schomburgkii* |
| N1 | Non-polluted | 15 | 20 | 10.23731 | -61.61402 | 32.2 | 3.2 | 230.6 | 262.1 | 149.5 | 0.11 | 6.97 |  |
| N2 | Non-polluted | 21 | 19 | 10.20173 | -61.63305 | 26.5 | 1.22 | 544 | 555 | 351 | 0.2 | 6.64 |  |
| P1 | Polluted | 15 | 14 | 10.19644 | -61.63309 | 27.8 | 2.09 | 2530 | 2643 | 1644.5 | 1.3 | 7.07 | *Astyanax bimaculatus* |
| P2 | Polluted | 20 | 10 | 10.17080 | -61.68113 | 29.7 | 1 | 11.409 | 13573 | 7091.5 | 5.05 | 7.47 | *Anablepsoides hartii* |

## **Appendix Table 2:** Results from 2019 analysis of colour intensity, with A) results from the original MANCOVA comparing pollution, population(pollution) and standard length as covariate across all 3 colour types. B) Post-Hoc ANCOVA results for each individual colour type. C) Pairwise comparisons between populations nested within pollution regime. Significant *P-*values are highlighted in bold.

| **A) MANCOVA** | | | | |
| --- | --- | --- | --- | --- |
| Factor | *F* | df | *P* | *η_P_^2^* |
| Standard Length | 2.098 | 3,72 | 0.108 | 0.08 |
| **Pollution** | **3.201** | **3,72** | **0.028** | **0.118** |
| Population(Pollution) | 1.513 | 9,222 | 0.144 | 0.058 |
| **B) Post-Hoc ANCOVAs** | | | | |
| Dependent variable | Factor | *F* | df | *P* |
| ΔE Orange | **Standard Length** | **4.273** | **1,74** | **0.042** |
|  | **Pollution** | **8.92** | **1,74** | **0.004** |
|  | Population(Pollution) | 2.322 | 3,74 | 0.082 |
| ΔE Iridescence | **Standard Length** | **5.219** | **1,74** | **0.025** |
|  | **Pollution** | **4.749** | **1,74** | **0.032** |
|  | Population(Pollution) | 1.854 | 3,74 | 0.145 |
| ΔE Black | Standard Length | 3.696 | 1,74 | 0.058 |
|  | **Pollution** | **5.728** | **1,74** | **0.019** |
|  | **Population(Pollution)** | **3.241** | 3,74 | **0.027** |
| C) Pairwise comparisons | | | | |
| Dependent variable | Comparison | Mean difference | Std. error | *P* |
| ΔE Orange | P1 vs P2 | -4.267 | 2.404 | 0.087 |
|  | N1 vs N2 | -1.957 | 1.966 | 0.323 |
| ΔE Iridescence | P1 vs P2 | 0.003 | 2.44 | 0.999 |
|  | N1 vs N2 | -1.475 | 1.996 | 0.462 |
| ΔE Black | P1 vs P2 | -1.877 | 1.785 | 0.296 |
|  | N1 vs N2 | -1.299 | 1.46 | 0.377 |

## **Appendix Table 3:** Mean L*a*b* values for each colour type across each population, as well as overall mean L*a*b* values for each pollution regime in 2018.

|  |  | **Orange** | | | **Iridescence** | | | **Black** | | |
| --- | --- | --- | --- | --- | --- | --- | --- | --- | --- | --- |
|  |  | **L*** | **A*** | **B*** | **L*** | **A*** | **B*** | **L*** | **A*** | **B*** |
| **Population** | **Pollution** | **A) Population** | | | | | | | | |
| N1 | Non-Polluted | 42.15208 | 13.61505 | 9.663242 | 53.77383 | 5.008927 | -2.44395 | 30.52955 | 7.483202 | 3.86489 |
| N2 | Non-Polluted | 42.18506 | 13.25713 | 12.63722 | 52.78607 | 4.782083 | 1.287822 | 30.93062 | 7.328527 | 4.717435 |
| P1 | Polluted | 40.47138 | 15.79339 | 9.6809 | 53.81847 | 4.536188 | 0.004696 | 32.44138 | 6.40957 | 5.477446 |
| P2 | Polluted | 35.54493 | 13.35172 | 8.269379 | 46.5545 | 4.860595 | -0.88545 | 27.90693 | 7.840807 | 4.444405 |
| PL | Pitch Lake | 33.91584 | 17.11489 | 13.62977 | 43.95077 | 7.844965 | -1.26472 | 26.71222 | 9.3266 | 4.753676 |
| **Pollution** | | **B) Pollution Regime** | | | | | | | | |
| Non-Polluted | | 42.17107 | 13.40898 | 11.37553 | 53.20512 | 4.87832 | -0.29535 | 30.76047 | 7.394147 | 4.355749 |
| Polluted | | 37.57347 | 14.35711 | 8.850593 | 49.66763 | 4.721563 | -0.50396 | 29.85027 | 7.22742 | 4.887137 |
| Pitch Lake | | 33.91584 | 17.11489 | 13.62977 | 43.95077 | 7.844965 | -1.26472 | 26.71222 | 9.3266 | 4.753676 |
|  |  |  |  |  |  |  |  |  |  |  |

## **Appendix Table 4:** Mean L*a*b* values for non-ornamentation body colouration for 5 randomly selected individuals per population. Means calculated both across populations and by pollution regime.

| **Population** | **Pollution** | **L*** | **A*** | **B*** |
| --- | --- | --- | --- | --- |
| N1 | Non-Polluted | 46.2 | 4.49 | 9.39 |
| N2 | Non-Polluted | 48.7 | 5.13 | 10.5 |
| P1 | Polluted | 48.9 | 4.52 | 11.9 |
| P2 | Polluted | 41.3 | 5.22 | 9.98 |
| PL | Pitch Lake | 37.5 | 6.03 | 10.1 |
| Non-Polluted | | 47.45 | 4.81 | 9.945 |
| Polluted | | 45.1 | 4.87 | 10.94 |
| Pitch Lake | | 37.5 | 6.03 | 10.1 |


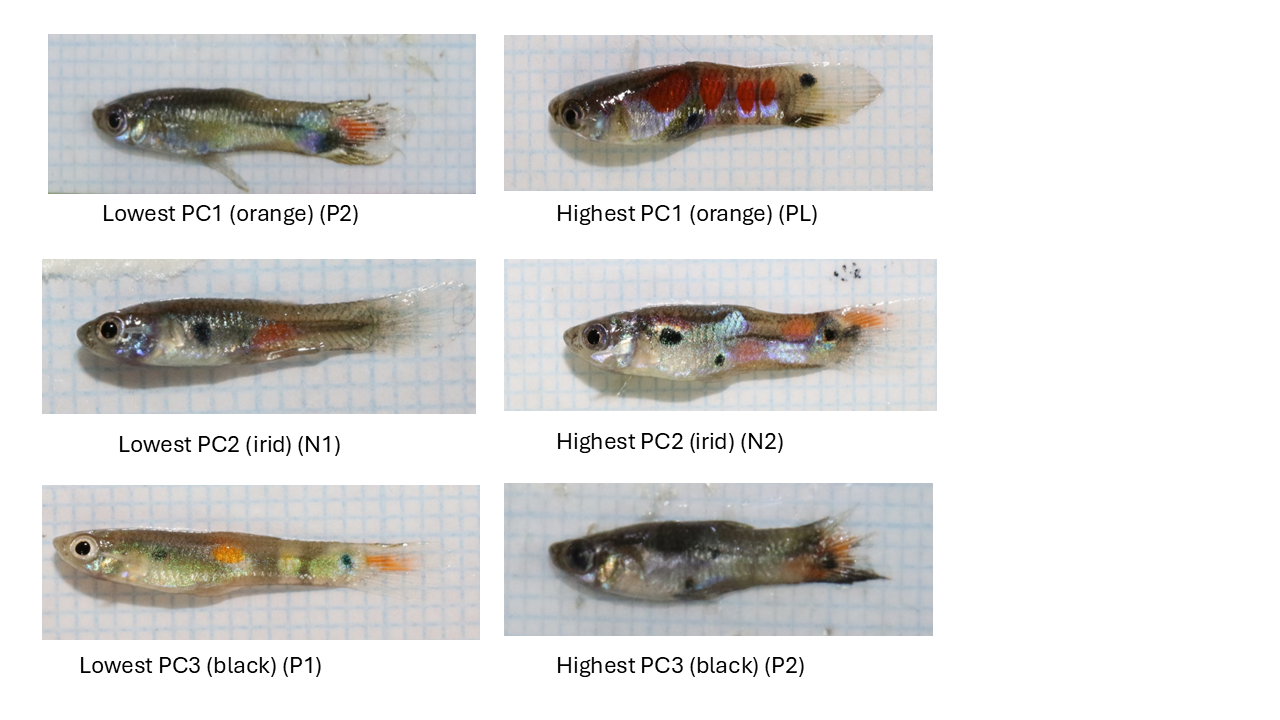

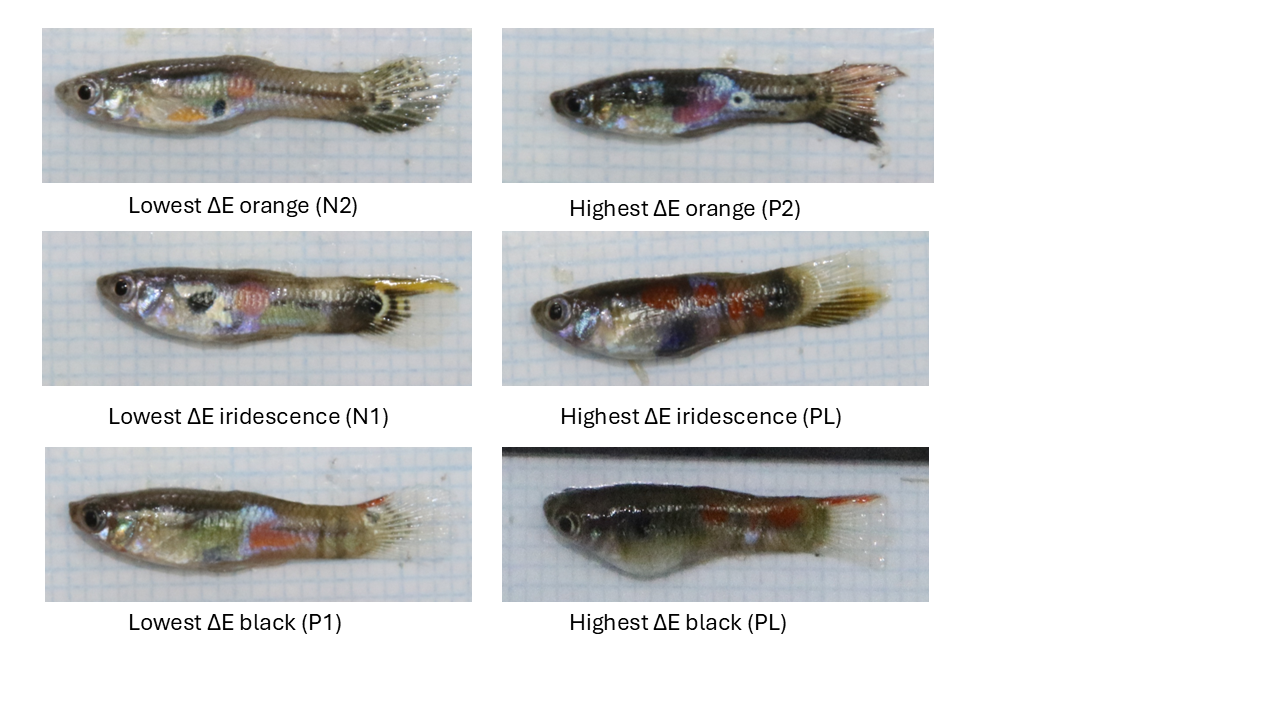


**Appendix Figure 1:** Examples of highest and lowest area and intensity values for all fish across all populations. The population of origin for each fish is indicated in brackets. Area, in terms of number and size of colour patches, is denoted by PC. Intensity is measured by ΔE with higher ΔE indicating greatest deviation from the maximum L*a*b* values.


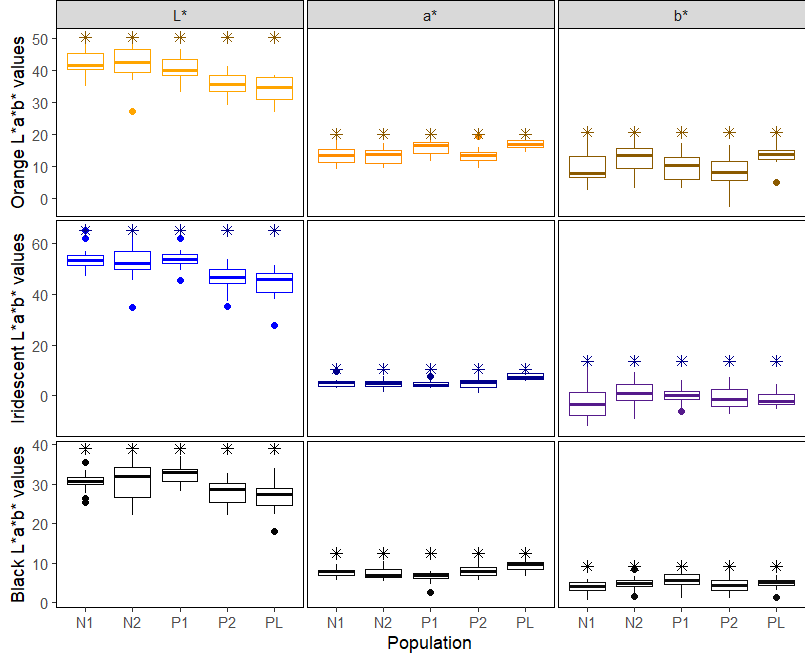


**Appendix Figure 2:** Breakdown of L*a*b* values for each population across all three colour types.


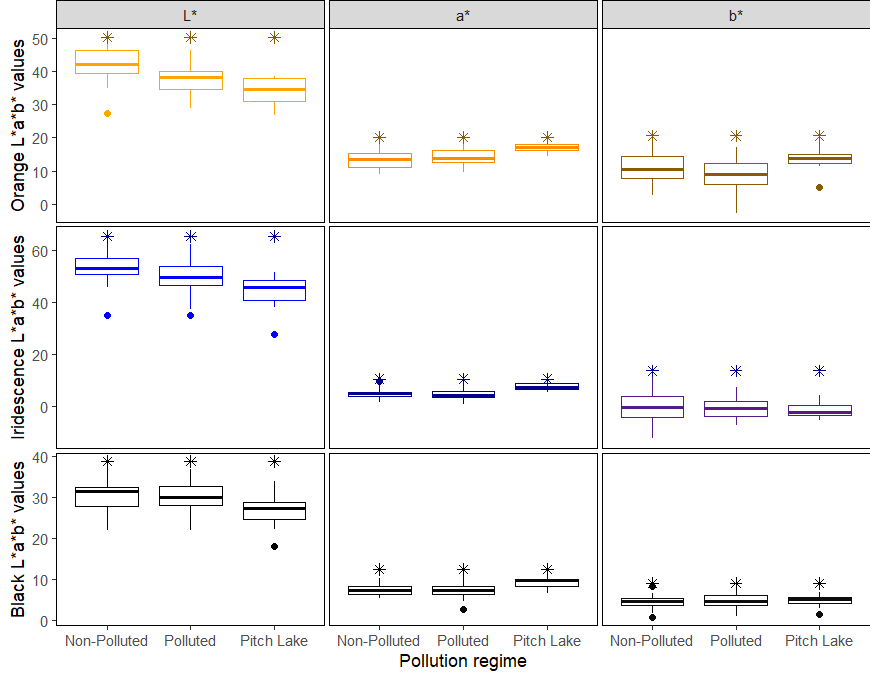


**Appendix Figure 3:** Breakdown of L*a*b* values for each pollution regime across all three colour types. “Polluted” in this instance refers to anthropogenic pollution.
